# Cross‐Cultural Adaptation and Validation of the Japanese Charité Alarm Fatigue Questionnaire (CAFQa) Among ICU Nurses and Physicians: A Multicentre Study

**DOI:** 10.1111/nicc.70576

**Published:** 2026-07-07

**Authors:** Tomoo Sato, Mitsuko Ishiseki, Yuki Kataoka, Hidehiro Someko, Hiroki Sato, Kota Minami, Takao Kaneko, Chikashi Takeda, Adam Crosby

**Affiliations:** ^1^ Department of Nursing, Faculty of Nursing Kindai University Sakai City, Osaka Japan; ^2^ Scientific Research WorkS Peer Support Group (SRWS‐PSG) Osaka Japan; ^3^ Adult and Child Health Care Nursing Region, Acute Care Nursing Division Kobe City College of Nursing Kobe City Japan; ^4^ Center for Postgraduate Clinical Training and Career Development Nagoya University Hospital Nagoya‐city Japan; ^5^ Center for Medical Education, Graduate School of Medicine Nagoya University Nagoya‐city Japan; ^6^ Department of Healthcare Epidemiology, Graduate School of Medicine Kyoto University Kyoto Japan; ^7^ Department of International and Community Oral Health Tohoku University Graduate School of Dentistry Sendai Japan; ^8^ Department of Internal Medicine Kyoto Min‐Iren Asukai Hospital Kyoto Japan; ^9^ Department of Internal Medicine Nagoya Tokushukai General Hospital Kasugai Japan; ^10^ Department of Healthcare Epidemiology Kyoto University Graduate School of Medicine/Public Health Kyoto Japan; ^11^ Faculty of Rehabilitation Kawasaki University of Medical Welfare Okayama Japan; ^12^ Medical Training Center Saga University Hospital Saga Japan; ^13^ Department of Rehabilitation Yamagata Prefectural Central Hospital Yamagata Japan; ^14^ Department of Pharmacoepidemiology, Graduate School of Medicine and Public Health Kyoto University Kyoto Japan; ^15^ Department of Anesthesia Kyoto University Hospital Kyoto Japan; ^16^ Kobe City College of Nursing Kobe City Japan

**Keywords:** alarm fatigue, alarm management, cross‐cultural adaptation, intensive care unit, questionnaire, reliability, validation

## Abstract

Alarm fatigue threatens patient safety in intensive care units (ICUs), yet no validated Japanese instrument exists. We cross‐culturally adapted the Charité Alarm Fatigue Questionnaire (CAFQa) into Japanese following COSMIN guidelines and evaluated it in 129 clinicians (103 nurses, 26 physicians) at five hospitals, with 102 retested. Confirmatory factor analysis supported the two‐factor structure (CFI = 0.922; RMSEA = 0.041; SRMR = 0.076), but the inter‐factor correlation was near zero (*r* = 0.05), suggesting that Alarm Stress and Alarm Coping behave as largely independent dimensions. Cronbach's alpha was 0.805 (Stress) and 0.649 (Coping); test–retest ICCs ranged 0.62–0.75. Alarm Stress correlated with job stress (*r* = 0.256) and insomnia (*r* = 0.369), whereas Alarm Coping related selectively to relational and environmental factors and to ICU experience. Given the orthogonal subscales, we recommend subscale‐level rather than total‐score interpretation.

## 1 Introduction

The ICU relies extensively on monitoring devices that generate alarms to alert healthcare professionals to changes in patients' conditions. However, 72%–99% of these alarms are false or do not require clinical intervention [1, 2, 3, 4]. The resulting alarm fatigue threatens patient safety by desensitising staff to critical alerts, with cases of patient deaths reported [5], and is associated with headaches, hearing impairment [6], sleep disturbance [7] and burnout [8] in healthcare professionals. Alarm fatigue is recognised as a National Patient Safety Goal in the United States [1] and consistently ranks among the top technology hazards [9]. Reliable measurement is a prerequisite for testing mitigation strategies. The Charité Alarm Fatigue Questionnaire (CAFQa) [10], a 9‐item instrument with two subscales (Alarm Stress and Alarm Coping), has been validated in German [10, 11], Dutch [12] and English [13], but no validated Japanese instrument exists.

Several features of the Japanese ICU environment make a context‐specific validation necessary rather than a simple translation. Under Japan's intensive‐care reimbursement criteria, eligible units must maintain a nurse‐to‐patient ratio of at least 1:2 at all times, so a small nursing team is continuously exposed to the full monitored‐alarm load [14]. Authority to set and adjust alarm thresholds is largely concentrated among physicians, leaving nurses, who respond to most alarms, with limited latitude to tailor settings. In addition, organisational alarm‐management protocols have penetrated Japanese ICUs unevenly. These conditions shape how alarm stress arises and how coping is enacted, and they may not be captured by versions developed in other health systems.

A validated Japanese instrument is intended to serve two complementary purposes: as an occupational‐health tool for monitoring staff burnout and mental‐health risk attributable to alarm exposure and as a patient‐safety metric, for example, as an outcome measure for alarm‐reduction interventions.

### 1.1 Aim

This study aimed to cross‐culturally adapt the CAFQa into Japanese and evaluate its reliability and validity among ICU nurses and physicians to provide a Japanese instrument suitable for occupational‐health and patient‐safety monitoring in ICUs.

## 2 Design and Methods

Following COSMIN guidance [15] (Table S1), we performed forward and backward translation, expert‐panel review (four physicians, four nurses, one translator), and cognitive interviews with seven ICU clinicians (two physicians, five nurses) to produce the Japanese CAFQa [16], then conducted a multicentre cross sectional survey with a test–retest component. Participants were nurses and physicians from ICUs at five hospitals. Concurrent measures were the NIOSH Generic Job Stress Questionnaire [17] and the Insomnia Severity Index (ISI) [18]. The CAFQa comprises five Alarm Stress items and four reverse‐scored Alarm Coping items on five‐point scales; higher scores indicate greater alarm fatigue. This study was approved by the institutional review boards of Kobe City College of Nursing (approval number: 25104‐04) and Kyoto University Graduate School and Faculty of Medicine (approval number: R5182‐1). All participants provided informed consent.

Structural validity was assessed by confirmatory factor analysis (CFA) using the unweighted least‐squares (ULS) estimator on polychoric correlations, appropriate for ordered‐categorical responses, with CFI, TLI, RMSEA and SRMR [10, 11, 12, 13, 19]. Internal consistency was indexed by Cronbach's alpha, McDonald's omega (total and hierarchical) and the mean inter‐item correlation (MIC). Because the CAFQa comprises two conceptually distinct subscales, validity analyses were planned at the subscale level; the total score was retained only as a descriptive summary for comparability with prior versions. We tested the hypothesis that, if Alarm Coping indexed individual alarm‐management competence, more experienced clinicians and physicians would show better (lower) coping scores. Convergent validity was examined using Pearson correlations, and exploratory known‐groups comparisons used Mann–Whitney tests. Following inspection of the near‐zero inter‐factor correlation, all primary analyses were interpreted at the subscale level. Analyses used R 4.6.0 (psych, lavaan, irr).

## 3 Results

**FIGURE 1:**
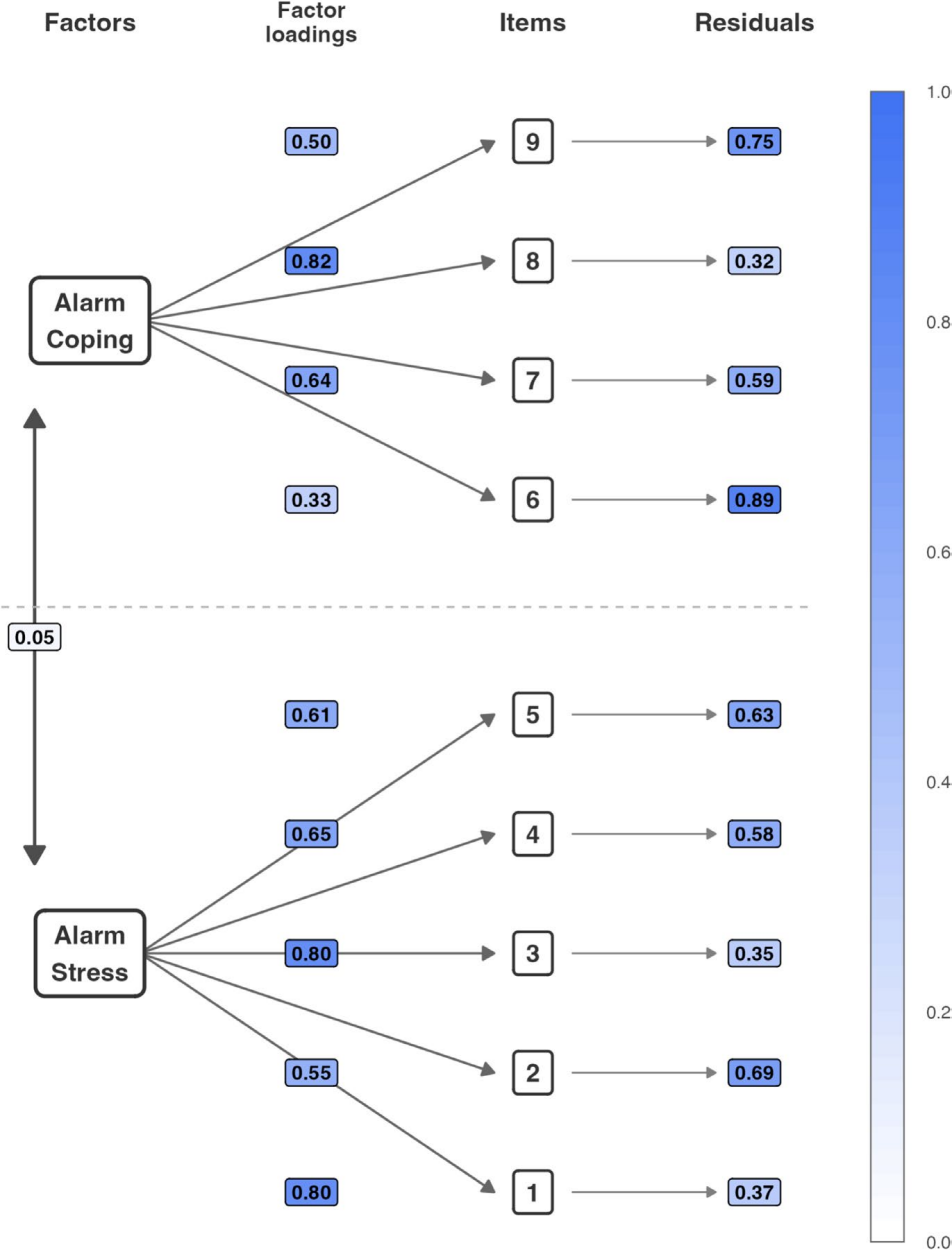
Structural diagram of the two factor model. The diagram displays the two‐factor model comprising Alarm Stress (Items 1–5) and Alarm Coping (Items 6–9). Values on the left represent standardised factor loadings, and values on the right represent residual variances for each item. The inter‐factor correlation between Stress and Coping was 0.05. The colour gradient on the right side of the figure represents the magnitude of residual variance, with darker shading indicating higher values. The model was estimated using the unweighted least squares (ULS) estimator on polychoric correlations. All factor loadings were significant (*p* < 0.001).

Of 129 clinicians (103 nurses, 26 physicians) across five hospitals, 102 were retested (Table S2). CFA supported the two‐factor structure (CFI = 0.922; TLI = 0.891; RMSEA = 0.041; SRMR = 0.076), with loadings of 0.55–0.80 for Alarm Stress and 0.33–0.82 for Alarm Coping (Figure 1; Table S4). The inter‐factor correlation was near zero (*r* = 0.05), suggesting that the subscales operate as essentially independent dimensions.

Item distributions and floor/ceiling effects are reported in Table 1. Cronbach's alpha was 0.805 (Alarm Stress), 0.649 (Alarm Coping) and 0.688 (total) (Table S3). McDonald's omega‐total was 0.82 and 0.67, respectively. For the full scale, omega‐hierarchical was 0.028 (95% CI 0.006–0.295) (Table S8). The MIC was 0.46 (SD 0.11) for Alarm Stress but 0.31 (SD 0.17) for Alarm Coping, within which Items 7–9 were moderately intercorrelated (*r* = 0.36–0.51) whereas Item 6 was nearly independent of them (r = 0.03 with Item 9) (Table S7). Test–retest ICCs ranged 0.62–0.75; the standard error of measurement was 1.91 (Alarm Stress) and 1.55 (Alarm Coping), corresponding to minimal detectable changes of 5.30 and 4.29, respectively (Table S3; Figure S1).

**TABLE 1:** Item descriptive statistics, factor loadings and floor/ceiling effects (*N* = 129).

Items	Content (abbreviated)	Mean (SD)	Skew	Loading	Floor %	Ceiling %
Alarm stress (items 1–5)						
1	Work performance impaired	0.42 (0.95)	−0.26	0.55–0.80	2.3	11.6
2	Physical symptoms	0.02 (1.17)	0.10		9.3	13.2
3	Reduced concentration	0.19 (0.97)	0.02		3.9	10.1
4	Workflow interrupted	0.69 (0.89)	−0.02		0.0	20.9
5	Confused by alarms	0.44 (0.99)	−0.34		3.1	13.2
Alarm coping (items 6–9, reverse‐scored)						
6	Protocols updated/shared	0.92 (0.83)	−0.82	0.33	0.8	22.5
7	Staff respond promptly	−0.44 (0.93)	0.06	0.33–0.82	13.2	0.8
8	Monitor information clear	−0.53 (0.90)	0.36	0.82	12.4	1.6
9	Limits customised	−1.03 (0.81)	0.58		29.5	0.0

*Note:* Items scored so that higher = greater alarm fatigue (coping items 6–9 reverse‐scored). Floor/ceiling = % selecting the minimum/maximum option; ≥ 15% indicates an effect (item 4 ceiling; Item 6 ceiling; Item 9 floor). ULS estimator on polychoric correlations. Inter‐factor *r* = 0.05. Cronbach's alpha: Stress 0.805, Coping 0.649. McDonald's omega‐total: Stress 0.82, Coping 0.67; full‐scale omega‐hierarchical 0.028 (95% CI 0.006–0.295). Mean inter‐item correlation: Stress 0.46, Coping 0.31 (Item 6 with Item 9, *r* = 0.03).

Convergent validity was evaluated at the subscale level (Table S5). Alarm Stress correlated with the NIOSH total (*r* = 0.256) and the ISI (*r* = 0.369; both *p* < 0.01), whereas Alarm Coping correlated with neither (*r* = 0.094 and 0.063) and was instead selectively related to workplace relational and environmental factors (*r* = 0.218–0.233) and ICU experience (*r* = 0.267, *p* = 0.002) (Figure S2C). For comparison with prior versions, the total‐score correlations (NIOSH *r* = 0.261; ISI *r* = 0.338; Figure S2A,B) are reported descriptively only and were driven almost entirely by Alarm Stress.

In exploratory known‐groups comparisons, physicians scored higher than nurses on Alarm Coping (*p* = 0.003), and clinicians with ≥ 4 years' experience scored higher than those with 1–3 years (*p* = 0.044), in both cases indicating poorer, not better, coping; total scores did not differ by occupation (*p* = 0.986). The experience difference did not persist within occupational strata (nurses *p* = 0.281; physicians *p* = 0.206), and the ≥ 4‐year group contained more physicians, suggesting confounding by occupation (Table S6).

## 4 Discussion

The near‐zero inter‐factor correlation (*r* = 0.05) is the most striking finding of this validation. It contrasts with the German (0.40–0.44) [10, 11] and Dutch (0.46) [12] versions but matches the independent English validation (0.12) [13], and was corroborated by a near‐zero omega‐hierarchical for the total score. Because all versions used the same estimator and two‐factor structure, a methodological artefact is unlikely, and the replication in English argues against a translation‐specific cause. The subscales differ in referent: Alarm Stress captures an individual affective response, whereas Alarm Coping aggregates distinct features of the unit alarm environment (protocols, responsiveness, monitor clarity and customisation) that need not co‐occur. Such constructs can be largely independent: a well‐run unit may still leave staff stressed, and vice versa. Although prior validations defaulted to a summed total score, our results replicate the low correlation and quantify it with omega‐hierarchical. We therefore recommend, for the first time at the operational level, that the two subscales be scored and interpreted separately.

Two indices fell short of conventional thresholds: TLI (0.891) and Alarm Coping reliability (alpha 0.649). Rather than reassurance, the recurrence of below‐threshold Coping reliability across the German (0.46–0.57) [10, 11], Dutch (0.46) [12], English (0.68) [13] and Japanese versions points to a structural limitation of the construct. This is quantitatively evident in the inter‐item structure: the Coping items split into an operational/technical cluster (Items 7–9, *r* = 0.36–0.51) and a near‐isolated organisational item (Item 6, *r* = 0.03 with Item 9) (Table S7). Consistent with this, Item 6 (organisational protocol updating) is the only Coping item with a positive mean across all language versions, oppositely valenced to the others (Table S9), indicating that it taps a distinct organisational facet. Such a pattern is the signature of a multifaceted, formative‐like construct, for which a conventional alpha threshold is not the appropriate criterion; incremental indices such as TLI are also deflated when average inter‐item correlations are low, as here. The Coping subscale therefore warrants conceptual refinement rather than mere item deletion.

Contrary to our a priori expectation that alarm‐management competence would improve with experience, more‐experienced clinicians reported poorer Alarm Coping in the pooled sample, but this difference did not persist within occupational strata. Because the small physician subgroup was almost entirely composed of experienced clinicians, occupation and experience could not be disentangled. Rather than a decline in individual coping ability, this pattern, exploratory and underpowered, is consistent with Alarm Coping reflecting appraisal of the unit alarm environment rather than individual skill, since a competence‐based account predicts the opposite direction. In the Japanese setting, where alarm‐setting authority is concentrated among physicians, clinicians who configure alarms may appraise the unit alarm environment more critically; this remains a hypothesis to be tested in larger, occupationally balanced samples.

These findings have practical implications. Because Alarm Stress and Alarm Coping are independent, a single intervention is unlikely to move both: system‐level changes to the alarm environment (updated protocols, default settings, monitor configuration) should register on Alarm Coping, whereas the affective burden captured by Alarm Stress may instead require workload reduction and mental‐health or individual‐support measures. The two subscales thus serve the instrument's dual purpose (Alarm Stress as an occupational‐health signal and Alarm Coping as a system and patient‐safety indicator) and should be tracked separately when evaluating alarm‐reduction programmes.

Limitations include a 32.6% response rate (possible non‐response bias), the small physician subgroup, the structural limitations of the Coping subscale and the single‐country cross sectional design. The near‐zero inter‐factor correlation means the total score lacks a coherent unidimensional interpretation and should be used, if at all, only as a rough descriptive summary; the prioritisation of subscale‐level interpretation was confirmed post hoc. Whether the low correlation reflects the construct, this sample or residual translational effects cannot be resolved here; formal measurement‐invariance testing across languages and settings is required before cross‐cultural comparability is established.

## 5 Conclusion

The Japanese CAFQa measures two largely independent dimensions of alarm fatigue. The Alarm Stress subscale showed acceptable reliability and coherent convergent validity, whereas the Alarm Coping subscale showed below‐threshold internal consistency and structural heterogeneity, consistent with all previous language versions and indicating a limitation of the coping construct. Because the two dimensions were essentially independent, they should be scored and interpreted separately rather than summed. Pending further evaluation of measurement invariance, responsiveness and interpretable thresholds, the instrument is best suited to subscale‐level, group‐level comparisons rather than individual assessment.

## Author Contributions

**Tomoo Sato:** conceptualization, methodology, validation, formal analysis, investigation, data curation, writing – original draft, writing – review and editing, and supervision. **Mitsuko Ishiseki:** conceptualization, methodology, investigation, data curation. **Yuki Kataoka:** methodology, validation, formal analysis, writing – review and editing, and supervision. **Hidehiro Someko:** methodology, validation, formal analysis, writing – review and editing, and supervision. **Hiroki Sato:** methodology, investigation, and writing – review and editing. **Kota Minami:** methodology, investigation, and writing – review and editing. **Takao Kaneko:** methodology, investigation, and writing – review and editing. **Adam Crosby:** writing – review and editing. **Chikashi Takeda:** investigation, and writing – review and editing.

## Funding

This work was supported by the JSPS KAKENHI Grant 25K13969.

## Ethics Statement

This study was conducted in accordance with the Declaration of Helsinki. The study protocol was approved by the institutional review board of Kobe City College of Nursing on November 28, 2024 (approval number: 25104‐04) and Kyoto University Graduate School and Faculty of Medicine on October 27, 2025 (approval number: R5182‐1). Participation was voluntary and anonymous. All participants provided informed consent by checking a consent box on the online survey prior to participation.

## Conflicts of Interest

The authors declare no conflicts of interest.

## Supporting information


**Data S1:** Supporting Information.

**Table S1:** COSMIN reporting checklist (version 2.0).
**Table S2:** Demographic characteristics of participants.
**Table S3:** Reliability of the Japanese CAFQa: Internal consistency, test–retest reliability and measurement error.
**Table S4:** Confirmatory factor analysis.
**Table S5:** Correlation matrix for convergent validity of the Japanese CAFQa.
**Table S6:** Comparison of Japanese CAFQa scores by occupation and clinical experience.
**Table S7:** Subscale inter‐item correlation matrices of the Japanese CAFQa (*N* = 129).
**Table S8:** McDonald's omega reliability of the Japanese CAFQa (*N* = 129).
**Table S9:** Cross‐version comparison of alarm coping item means on the common −2 to +2 scale.
**Figure S1:** Bland–Altman plots for test–retest agreement of the Japanese CAFQa (*n* = 102).
**Figure S2:** Scatter plots for convergent and hypothesis‐specific validity of the Japanese CAFQa.

## Data Availability

Due to the nature of this research, participants of this study did not agree for their data to be shared publicly, so supporting data are not available.
