# Supplementary material for: Cross‐Cultural Adaptation and Validation of the Japanese Charité Alarm Fatigue Questionnaire (CAFQa) Among ICU Nurses and Physicians: A Multicentre Study
Source: Nurs Crit Care. 2026 Jul 7;31(4):e70576. doi: 10.1111/nicc.70576 (PMC13339743; doi:10.1111/nicc.70576)
Supplement: Supplementary file 1 — Data S1: Supporting Information. [file NICC-31-0-s002.docx]

**日本語版アラーム疲労質問票**

本質問票は、ICU（集中治療室）に勤務する医師および看護師の方を対象に、アラーム疲労（医療機器　　　　　アラームによる疲労）を把握することを目的にしています。

以下の各項目では、ICU内で日常的に経験するアラームに関する疲労の程度や、アラームへの対処状況についてお尋ねします。あなたご自身の状況や考えに最も当てはまるものを**1つだけ**お選びください。

|  | とても  そう思う | そう思う | 部分的にそう思う | そう  思わない | 全くそう思わない |
| --- | --- | --- | --- | --- | --- |
| 1. 病棟内のアラームが多すぎると、業務のパフォーマンスやモチベーションが低下する。^1^ | □ | □ | □ | □ | □ |
| 1. アラームが多すぎると、緊張や頭痛、睡眠障害などの　身体的な症状が現れる。^1^ | □ | □ | □ | □ | □ |
| 1. アラームによって集中力や注意力が低下する。^1^ | □ | □ | □ | □ | □ |
| 1. 自分の担当患者や隣接する患者のアラーム、または緊急アラームが頻繁に業務を中断させる。^1^ | □ | □ | □ | □ | □ |
| 1. アラームに惑わされることがある。^1^ | □ | □ | □ | □ | □ |
| 1. 自分の病棟では、アラーム対処に関する手順書が定期的に更新され、全スタッフと共有されている。*^2^ | □ | □ | □ | □ | □ |
| 1. 自分の病棟では、アラームに迅速かつ適切に対応している。*^2^ | □ | □ | □ | □ | □ |
| 1. 病棟フロアとセントラルモニターに使用されている音声と視覚的なモニターアラームは、患者、病室、緊急度を明確に知らせるようになっている。*^2^ | □ | □ | □ | □ | □ |
| 1. アラームの設定値は、患者の臨床症状に基づいて定期的に調整している（例：冠動脈バイパス術後の血圧の上限値）。*^2^ | □ | □ | □ | □ | □ |

^1^因子1の項目（アラームストレス尺度）

^2^因子2の項目（アラーム対処尺度）

**＊**印の付いた項目6～9（アラーム対処）はアラーム管理に関する肯定的な内容の質問であるため、

**逆転処理（4 − 素点）**を行い、すべての項目において得点が高いほどアラーム疲労が強いことを示す。

**各項目4点満点**で、CAFQa合計スコアの範囲は0～36点（アラームストレスサブスケールは0～20点、　　　　　アラーム対処サブスケールは0～16点）である。


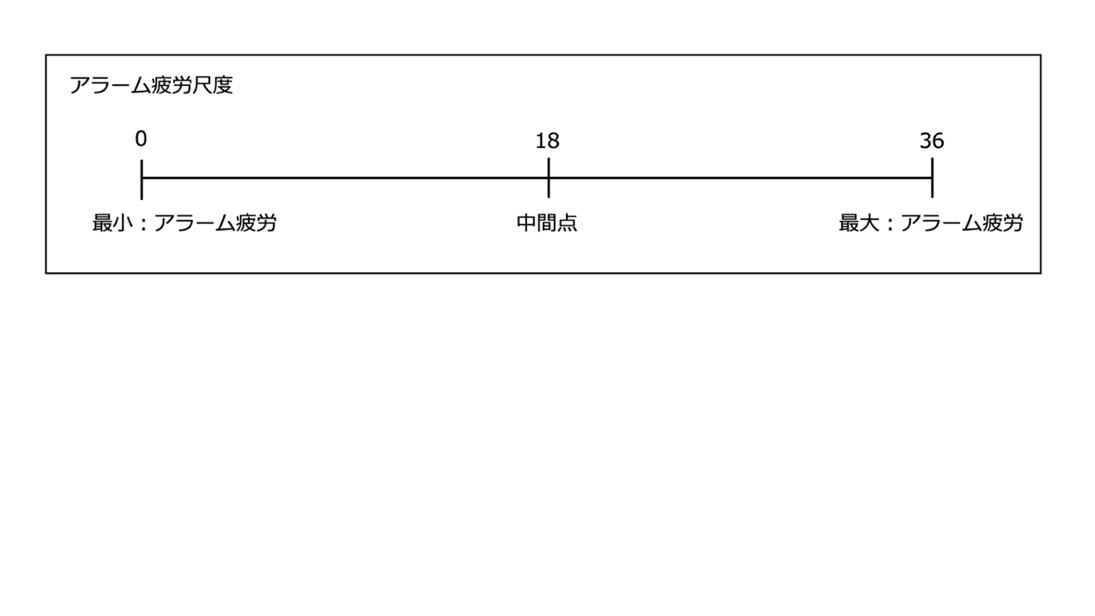


原典：Wunderlich, M.M., Amende-Wolf, S., Krampe, H. et al. A brief questionnaire for measuring alarm fatigue in nurses and

physicians in intensive care units. Sci Rep 13, 13860 (2023). <https://www.nature.com/articles/s41598-023-40290-7>

クリエイティブ・コモンズ・ライセンス（CC BY 4.0）：<https://creativecommons.org/licenses/by/4.0/deed.ja>

本質問票は、上記原著論文のオリジナル英語版を日本語に翻訳したものである。

本質問票をご利用の際は、論文の引用元を記載してください。

ご質問がございましたら、お気軽に連絡ください:[tomoosato0315@gmail.com](mailto:tomoosato0315@gmail.com)
