# Supplementary material for: Cross‐Cultural Adaptation and Validation of the Japanese Charité Alarm Fatigue Questionnaire (CAFQa) Among ICU Nurses and Physicians: A Multicentre Study
Source: Nurs Crit Care. 2026 Jul 7;31(4):e70576. doi: 10.1111/nicc.70576 (PMC13339743; doi:10.1111/nicc.70576)
Supplement: Supplementary file 2 — Table S1: COSMIN reporting checklist (version 2.0). Table S2: Demographic characteristics of participants. Table S3: Reliability of the Japanese CAFQa: Internal consistency, test–retest reliability and measurement error. Table S4: Confirmatory factor analysis. Table S5: Correlation matrix for convergent validity of the Japanese CAFQa. Table S6: Comparison of Japanese CAFQa scores by occupation and clinical experience. Table S7: Subscale inter‐item correlation matrices of the Japanese CAFQa (N = 129). Table S8: McDonald's omega reliability of the Japanese CAFQa (N = 129). Table S9: Cross‐version comparison of alarm coping item means on the common −2 to +2 scale. Figure S1: Bland–Altman plots for test–retest agreement of the Japanese CAFQa (n = 102). Figure S2: Scatter plots for convergent and hypothesis‐specific validity of the Japanese CAFQa. [file NICC-31-0-s001.docx]

**Supplementary Materials**

**Supplementary Table 1. COSMIN Reporting Checklist (Version 2.0)**

| **General Reporting recommendations relevant for all studies on measurement properties** | | | |  |
| --- | --- | --- | --- | --- |
| **Item Number** | **Item Name** | **Item Description** | **Page** |  |
| **Report section: Title** | |  |  |  |
| T1 | Patient Reported Outcome Measure (PROM) | The name of the PROM instrument(s) (and version if relevant) being studied | Title page |  |
| T2 | Measurement Property (MP) | What MPs are being studied or more generally, that MPs are being studied (if there are many properties being investigated, for example) | Title page |  |
| T3 | Study sample | General description of relevant study sample characteristics (e.g., condition of interest, language) and also any intervention or exposure (e.g., treatments) if applicable. | Title page |  |
| **Report section: Abstract** | |  |  |  |
| A1 | PROM | The name of the PROM instrument(s) (and version if relevant) being studied (i.e. the SF-36 or SF-12; language version) or if it concerns an item bank (e.g., PROMIS instruments). The type of instrument (e.g. a self reported questionnaire or interview). | Page 1 |  |
| A2 | Measurement Property | What MPs are being studied or more generally, that MPs are being studied (if there are many properties being investigated, for example) | Page 1 |  |
| A3 | Design | The type of study being used to test the properties (e.g., test-retest design, longitudinal study, cohort, cross sectional, case series, randomized etc.). Other details of the study design if relevant (intervention/exposure, description of comparison instruments, outcomes other than PROMs). | Page 1 |  |
| A4 | Sample | Inclusion / exclusion criteria. General description of relevant study sample characteristics (e.g., condition of interest, geographic location, language, other relevant demographic and baseline characteristics) | Page 1 |  |
| A5 | Methods | A brief description of the methods for investigating each MP including statistical analyses | Page 1 |  |
| A6 | Results | The main results for all MPs investigated reporting statistics for each result with measures of precision where appropriate. | Page 1 |  |
| A7 | Discussion/Conclusions | A brief description of the results in the context of existing evidence, main strengths and drawbacks and the need for future research on the PROM(s) investigated. | Page 1 |  |
| **Report section: Introduction** | |  |  |  |
| I1 | Name and describe the PROM of interest | Specify the name, type, language, and version of the PROM being investigated and how it was developed. Describe the construct the PROM aims to measure and its subscales; describe the structure of the PROM (e.g., the number of factors, the number of items, scoring algorithm); describe relevant instructions (like time period), and number or type of response categories. State whether the PROM is based on a reflective or formative model.  Note: This information may also appear in the methods section in greater detail. | p.2 |  |
| I2 | Target population | Describe the specific target population that the PROM was designed for. The authors need to provide the appropriate and necessary characteristics of this population. | p.2 |  |
| I3 | Citation for the original development of the PROM | The citation for the original development paper(s) should be provided and other highly relevant citations related to the quality of the specific PROM under investigation. | p.2 |  |
| I4 | State of Knowledge & Rationale | A description of the current scientific knowledge (what is known) regarding the MPs of? the PROM under investigation. The authors should provide a literature review or refer to a recent review of all existing evidence of the specific version (e.g., language, short form) of the PROM and explain why the new study is necessary and important. The rational for the current proposed study should be given. | p.2 |  |
| I5 | Definitions | Specialized terms should be defined or explained. | p.2 |  |
| I6 | Objectives and Hypotheses | State the specific objective(s) of the research and hypotheses related to the specific PROM under investigation. | p.3 |  |
| **Report section: General Methods** | |  |  |  |
| GM1 | Study Design | State the key elements of the study design | p.3 |  |
| GM2 | Participants | State how the participants were chosen; the inclusion and exclusion criteria. (e.g., if a PROM for a specific condition, then the eligibility and selection criteria should reflect this). | p.3 |  |
| GM3 | PROM administration | An explicit description of how and when the PROM(s) were administered (e.g., in what setting) including data collection devices/system used (e.g. paper based, electronic administration / ePRO) should be provided. | p.3 |  |
| GM4 | Data collection procedures | Provide information about other data collection, exposure methods (e.g., allocation to interventions) and time points / follow-up points. | p.3 |  |
| GM5 | Power/sample size calculation | Provide a power calculation for all MP analyses. Alternatively, if a rule of thumb is used, state it and the source/citation. | N/A |  |
| GM6 | Statistical analyses | Statistical analyses and tests corresponding to all hypotheses or objectives for all MPs should be reported. Where appropriate, a cut-off for statistical significance should be reported (e.g., p-value less than 0.05). A description of all statistics to be used to estimate the magnitude and direction of effect should also be reported, together with measures of variability or precision. Report statistical package used. | p.3–4 |  |
| GM7 | Missing data | State approaches or plan for dealing with missing data. | N/A |  |
| GM8 | Post hoc analysis | The report should specify analyses that used data after the data collection period concluded (i.e., if the analyses were post hoc; secondary data analyses) and describe the rationale for any post hoc analyses. | p.4,7 |  |
| **Report section: General Results** | |  |  |  |
| GR1 | Missing data | The amount and reasons for missing data should be explained for all analyses for all PROMs (or other outcome measurement instruments) and relevant groups. | N/A |  |
| GR2 | Participant/patient Characteristics | The study patients’ characteristics should be described, including baseline PROM scores. | p.4 |  |
| GR3 | Sample size | If one study contained analyses using different sample sizes, the authors should report the sample size for each analysis. | p.4 |  |
| **Report section: Discussion** | |  |  |  |
| D1 | MP evidence | Per measurement property the authors should compare the result to the criteria for good measurement properties (e.g., COSMIN criteria)[27], and determine if the specific MP is sufficient or not. Note: This information may also appear in the results section in greater detail in a table for example. | p.6–7 |  |
| D2 | Practical relevance | The authors need to discuss the practical relevance of the findings. | p.6–7 |  |
| D3 | Strengths and limitations | Strengths and limitations of the study should be discussed. For example, discuss if there were any significant potential biases in the study that could have impacted the results. | p.7 |  |
| D4 | Generalizability | Generalizability issues related to the PROM results should be discussed. For example, discuss if the results could be generalized to other populations given the sample studied. | p.5–7 |  |
| D5 | Instrument changes | Discuss the need for modifications to the existing PROM or new PROM development. If you conclude that one of the measurement properties is insufficient, you could suggest some modification, or if it is really poor, you could suggest stopping use of the PROM (in the specific population or in general). | p.6–7 |  |
| D6 | Future Research | Report specifically the type of research needed to answer new questions arising out of these findings for the particular MP and PROM investigated. | p.7 |  |
| **Report section: Conclusions** | |  |  |  |
| C1 | Conclusions | State the overall conclusions for each MP and of the use PROM investigated. | p.7–8 |  |
| **Report section: Other information** | |  |  |  |
| O1 | Conflict of Interest | State any relevant conflict of interest related to the PROM under investigation (e.g., an author being the PROM developer, funding body etc). | Title page |  |

Gagnier JJ, de Arruda GT, Terwee CB, Mokkink LB; Consensus group. COSMIN reporting guideline for studies on measurement properties of patient-reported outcome measures: version 2.0. Qual Life Res. 2025 Jul;34(7):1901-1911. doi: 10.1007/s11136-025-03950-x. Epub 2025 Mar 28. PMID: 40153128

**Supplementary Table S2. Demographic Characteristics of Participants**

| **Characteristic** | **Value** |
| --- | --- |
| **N** | 129 |
| **Age (years)** | 33.1 (9.6) |
| **Gender, Female** | 93 (72.1) |
| **Occupation experience (years)** | 10.2 (8.7) |
| **ICU experience (years)** | 6.8 (6.5) |
| **Level of education** |  |
| **Vocational/Junior college** | 44 (34.1) |
| **University** | 66 (51.2) |
| **Master** | 8 (6.2) |
| **Doctoral** | 11 (8.5) |
| **Occupation** |  |
| **Nurse** | 103 (79.8) |
| **Doctor** | 26 (20.2) |
| **CAFQa Total** |  |
| **All** | 18.7 (4.5) |
| **Doctor** | 18.8 (4.0) |
| **Nurse** | 18.7 (4.7) |
| **Alarm Stress subscale (Items 1-5)** |  |
| **All** | 11.8 (3.7) |
| **Doctor** | 10.7 (3.8) |
| **Nurse** | 12.0 (3.7) |
| **Alarm Coping subscale (Items 6-9)*** |  |
| **All** | 6.9 (2.4) |
| **Doctor** | 8.1 (2.0) |
| **Nurse** | 6.6 (2.5) |

Table legend. Continuous variables are presented as mean (SD); categorical variables are presented as n (%).

CAFQa = Charité Alarm Fatigue Questionnaire.

Values are presented as mean (SD) for continuous variables and n (%) for categorical variables. ICU experience includes experience at previous institutions. CAFQa total scores range from 0 to 36, Alarm Stress subscale scores from 0 to 20, and Alarm Coping subscale scores from 0 to 16. Higher scores indicate greater alarm fatigue. *Items 6–9 (Alarm Coping) are reverse-scored; higher reversed scores indicate poorer alarm coping.

**Supplementary Table S3. Reliability of the Japanese CAFQa: Internal Consistency, Test–Retest Reliability, and Measurement Error**

| **Scale** | **N_items** | **Cronbach’s alpha** | **ICC** | **SEM** | **MDC** |
| --- | --- | --- | --- | --- | --- |
| **CAFQa Total** | 9 | 0.688 | 0.709 (0.597–0.794) | 2.52 | 6.98 |
| **Alarm Stress** | 5 | 0.805 | 0.753 (0.654–0.826) | 1.91 | 5.30 |
| **Alarm Coping** | 4 | 0.649 | 0.616 (0.480–0.724) | 1.55 | 4.29 |

Table legend. ICC = intraclass correlation coefficient; SEM = standard error of measurement; MDC = minimal detectable change at the 95% confidence level, calculated as 1.96 × √2 × SEM.

Cronbach's alpha ≥ 0.70 was considered acceptable for internal consistency. ICC ≥ 0.60 was considered indicative of acceptable test–retest reliability. Test–retest reliability was assessed in 102 participants who completed the CAFQa approximately two weeks after the initial administration.

**Supplementary Table S4. Confirmatory Factor Analysis**

| **Index** | **Value** | **Criterion** |
| --- | --- | --- |
| **Chi-square** | 31.6 |  |
| **df** | 26 |  |
| **p-value** | 0.0057 |  |
| **CFI** | 0.922 | >= 0.90 |
| **TLI** | 0.891 | >= 0.90 |
| **RMSEA** | 0.041 | <= 0.08 |
| **RMSEA 90% CI lower** | 0.000 |  |
| **RMSEA 90% CI upper** | 0.085 |  |
| **SRMR** | 0.076 | <= 0.08 |

Table legend. CFA = Confirmatory Factor Analysis; CFI = comparative fit index; TLI = Tucker-Lewis index; RMSEA = root mean square error of approximation; CI = confidence interval; SRMR = standardized root mean square residual.

Acceptable model fit was defined as CFI ≥ 0.90, TLI ≥ 0.90, RMSEA ≤ 0.08, and SRMR ≤ 0.08. The two-factor model comprised Alarm Stress (Items 1–5) and Alarm Coping (Items 6–9).

**Supplementary Table S5. Correlation Matrix for Convergent Validity of the Japanese CAFQa**

| **Variable** | **1** | **2** | **3** | **4** | **5** | **6** | **7** | **8** | **9** | **10** | **11** | **12** |
| --- | --- | --- | --- | --- | --- | --- | --- | --- | --- | --- | --- | --- |
| **1. Alarm Stress** | - | 0.041 | 0.845 | 0.274 | 0.164 | 0.200 | 0.219 | -0.138 | 0.154 | 0.162 | 0.256 | 0.369 |
| **2. Alarm Coping** |  | - | 0.569 | -0.069 | -0.131 | -0.083 | 0.218 | 0.224 | 0.233 | 0.231 | 0.094 | 0.063 |
| **3. CAFQa Total** |  |  | - | 0.189 | 0.065 | 0.12 | 0.297 | 0.006 | 0.252 | 0.257 | 0.261 | 0.338 |
| **4. NIOSH Workload** |  |  |  | - | 0.685 | 0.737 | 0.288 | -0.186 | 0.240 | 0.346 | 0.797 | 0.461 |
| **5. NIOSH Control** |  |  |  |  | - | 0.725 | 0.260 | -0.205 | 0.167 | 0.165 | 0.728 | 0.279 |
| **6. NIOSH Skill** |  |  |  |  |  | - | 0.357 | -0.09 | 0.158 | 0.280 | 0.797 | 0.349 |
| **7. NIOSH Interpersonal** |  |  |  |  |  |  | - | 0.242 | 0.265 | 0.282 | 0.548 | 0.626 |
| **8. NIOSH Environment** |  |  |  |  |  |  |  | - | 0.293 | 0.100 | 0.129 | -0.114 |
| **9. NIOSH Sup. Support** |  |  |  |  |  |  |  |  | - | 0.613 | 0.589 | 0.211 |
| **10. NIOSH Cow. Support** |  |  |  |  |  |  |  |  |  | - | 0.646 | 0.374 |
| **11. NIOSH Total** |  |  |  |  |  |  |  |  |  |  | - | 0.505 |
| **12. ISI Total** |  |  |  |  |  |  |  |  |  |  |  | - |

Table legend. CAFQa = Charité Alarm Fatigue Questionnaire; NIOSH = National Institute for Occupational Safety and Health Brief Job Stress Questionnaire; ISI = Insomnia Severity Index.

Values represent Pearson's correlation coefficients (r). Statistical significance was set at p < 0.05. Bold values indicate statistically significant correlations. Correlation strength was interpreted as weak (r < 0.3), moderate (r = 0.3–0.5), or strong (r > 0.5).

**Supplementary Table S6. Comparison of Japanese CAFQa Scores by Occupation and Clinical Experience**

| **Comparison** | **Scale** | **Group1** | **Group2** | **N group1** | **N group2** | **Median (IQR) group1** | **Median (IQR) group2** | **Effect r** |
| --- | --- | --- | --- | --- | --- | --- | --- | --- |
| **Occupation (Doctor vs Nurse)** | Alarm Stress | Doctor | Nurse | 26 | 103 | 10.0 (4.0) | 12.0 (4.0) | 0.173 |
| **Occupation (Doctor vs Nurse)** | Alarm Coping | Doctor | Nurse | 26 | 103 | 8.0 (2.0) | 7.0 (3.0) | 0.262 |
| **Occupation (Doctor vs Nurse)** | CAFQa total | Doctor | Nurse | 26 | 103 | 18.5 (3.8) | 19.0 (5.5) | 0.002 |
| **ICU experience (1-3yr vs 4+yr)** | Alarm Stress | 1-3 years | 4+ years | 54 | 75 | 11.5 (4.0) | 12.0 (4.0) | 0.092 |
| **ICU experience (1-3yr vs 4+yr)** | Alarm Coping | 1-3 years | 4+ years | 54 | 75 | 6.5 (3.0) | 7.0 (3.0) | 0.178 |
| **ICU experience (1-3yr vs 4+yr)** | CAFQa total | 1-3 years | 4+ years | 54 | 75 | 17.5 (6.0) | 19.0 (5.0) | 0.182 |
| **Occ. experience (1-3yr vs 4+yr)** | Alarm Stress | 1-3 years | 4+ years | 39 | 90 | 11.0 (4.5) | 12.0 (4.0) | 0.111 |
| **Occ. experience (1-3yr vs 4+yr)** | Alarm Coping | 1-3 years | 4+ years | 39 | 90 | 6.0 (4.0) | 7.5 (3.0) | 0.262 |
| **Occ. experience (1-3yr vs 4+yr)** | CAFQa total | 1-3 years | 4+ years | 39 | 90 | 17.0 (6.0) | 19.0 (5.0) | 0.239 |

Table legend. CAFQa = Charité Alarm Fatigue Questionnaire; Effect r = rank-biserial correlation coefficient as a measure of effect size; IQR = interquartile range; Occ. = Occupation.

Group comparisons were performed using the Mann-Whitney U test. ICU experience and occupation experience were dichotomized into 1–3 years and ≥4 years. Alarm Coping subscale scores reflect reverse-scored items (Items 6–9), with higher scores indicating poorer alarm coping. Statistical significance was set at p < 0.05.

**Supplementary Table S7. Subscale Inter-Item Correlation Matrices of the Japanese CAFQa (N = 129)**

**Panel A. Alarm Stress (Items 1–5)**

|  | **Item 1** | **Item 2** | **Item 3** | **Item 4** | **Item 5** |
| --- | --- | --- | --- | --- | --- |
| Item 1 | 1.000 | — | — | — | — |
| Item 2 | 0.491 | 1.000 | — | — | — |
| Item 3 | 0.616 | 0.486 | 1.000 | — | — |
| Item 4 | 0.478 | 0.277 | 0.523 | 1.000 | — |
| Item 5 | 0.474 | 0.288 | 0.447 | 0.554 | 1.000 |

**Panel B. Alarm Coping (Items 6–9, reverse-scored)**

|  | **Item 6** | **Item 7** | **Item 8** | **Item 9** |
| --- | --- | --- | --- | --- |
| Item 6 | 1.000 | — | — | — |
| Item 7 | 0.226 | 1.000 | — | — |
| Item 8 | 0.308 | 0.505 | 1.000 | — |
| Item 9 | 0.031 | 0.364 | 0.427 | 1.000 |

Table legend*.* Values are Pearson inter-item correlations (N = 129). The mean inter-item correlation (MIC) was 0.46 (SD 0.11; range 0.28–0.62) for Alarm Stress and 0.31 (SD 0.17; range 0.03–0.51) for Alarm Coping, indicating homogeneous content for Alarm Stress but marked heterogeneity for Alarm Coping. Within Alarm Coping, Items 7–9 were moderately intercorrelated (0.36–0.51) whereas Item 6 was nearly independent of them (e.g., r = 0.03 with Item 9). This pattern indicates that the Coping items capture partly distinct facets of unit alarm management rather than a single homogeneous trait. Coping items (6–9) were reverse-scored. The optimal MIC range is 0.15–0.50 (Clark & Watson, 1995).

**Supplementary Table S8. McDonald’s Omega Reliability of the Japanese CAFQa (N = 129)**

| **Scale** | **k (items)** | **Omega total (95% CI)** | **Omega hierarchical (95% CI)** |
| --- | --- | --- | --- |
| CAFQa Total | 9 | 0.777 (0.728–0.835) | 0.028 (0.006–0.295) |
| Alarm Stress | 5 | 0.816 (0.745–0.865) | — (single factor) |
| Alarm Coping | 4 | 0.670 (0.554–0.760) | — (single factor) |

Table legend. Omega total = proportion of total-score variance attributable to all common factors; omega hierarchical = proportion attributable to a single general factor shared across the two subscales. For the full scale, omega hierarchical was 0.03, indicating that a general alarm-fatigue factor accounted for only about 3% of total-score variance; this confirms, independently of the near-zero inter-factor correlation (r = 0.05), that the total score does not function as an interpretable unidimensional measure. Omega hierarchical is not meaningful for single-factor subscales. 95% confidence intervals were bootstrapped (B = 1000). Omega total ≥ 0.70 was considered acceptable (McNeish, 2018). Cronbach’s alpha for each scale is reported in Table S3.

**Supplementary Table S9. Cross-Version Comparison of Alarm Coping Item Means on the Common −2 to +2 Scale**

| **Alarm Coping item** | **German Study 1**  **(n = 265)** | **German Study 2**  **(n = 1212)** | **English (n = 248)** | **Japanese (n = 129)** |
| --- | --- | --- | --- | --- |
| Item 6 – protocols updated/shared | +0.77 | +1.10 | +0.02 | +0.92 |
| Item 7 – staff respond promptly | −0.32 | −0.39 | −0.87 | −0.44 |
| Item 8 – monitor information clear | −0.46 | −0.36 | −0.30 | −0.53 |
| Item 9 – limits customised | −0.38 | −0.35 | −0.33 | −1.03 |

Table legend. Item means are shown on the −2 to +2 metric in the fatigue direction (higher = poorer coping / more alarm fatigue; coping items reverse-scored). German values are from Wunderlich et al. (2024); English from Ruppel et al. (2026); Japanese from the present study. Across all versions, Item 6 (organisational protocol updating) is the only Alarm Coping item with a positive mean—that is, oppositely valenced to Items 7–9—indicating that it taps a distinct, organisational facet of alarm management rather than the individual/operational facet captured by the other items. Item-level means were not reported for the Dutch version (Gerardu et al., 2026); its confirmatory factor loading for Item 6 was 0.21, the lowest of all items, consistent with this pattern.

**Supplementary Figure S1. Bland-Altman Plots for Test–Retest Agreement of the Japanese CAFQa (n = 102)**


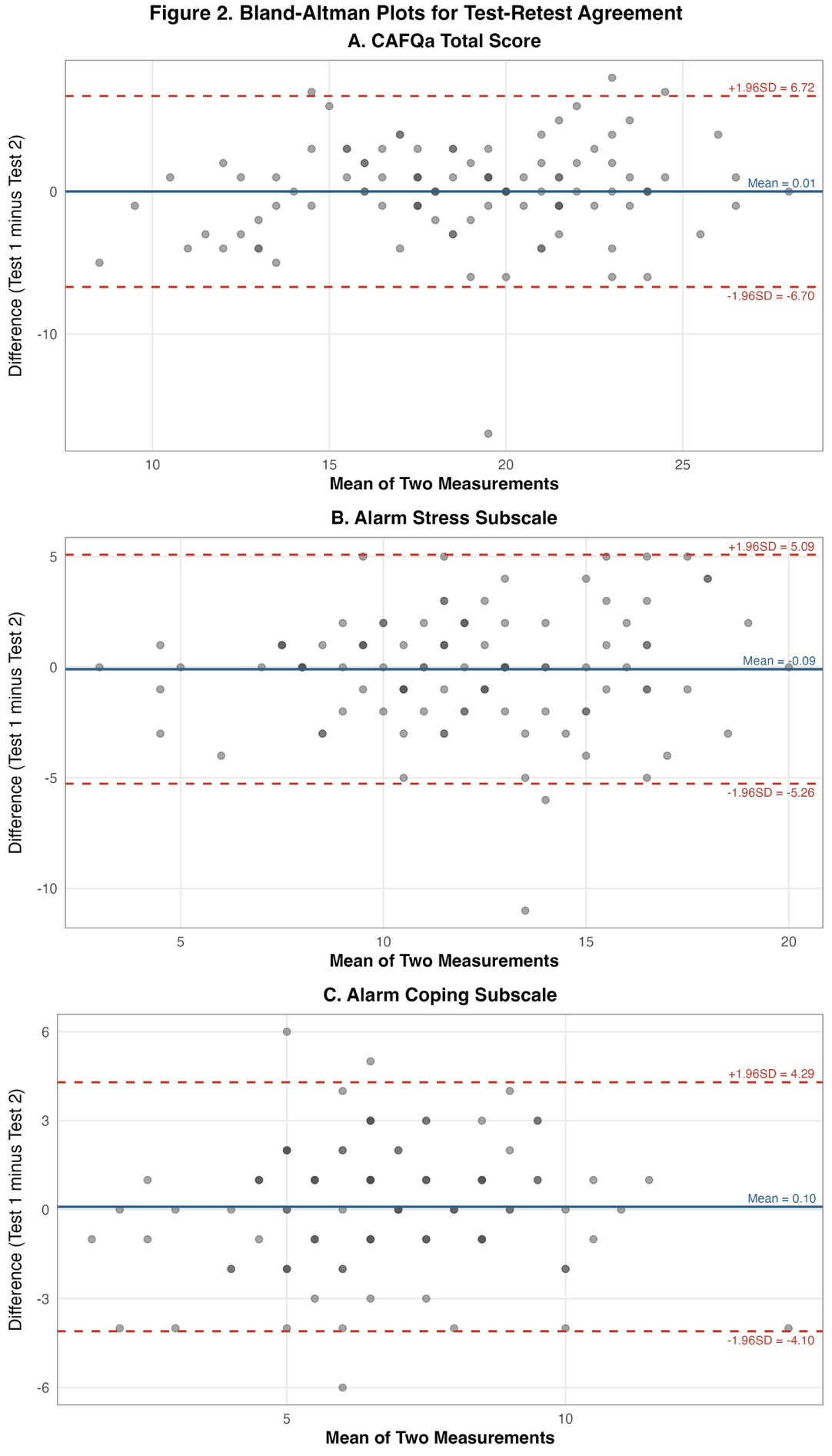


Figure legend. Panel A shows the CAFQa total score, Panel B shows the Alarm Stress subscale, and Panel C shows the Alarm Coping subscale. The x-axis represents the mean of the two measurements (Test 1 and Test 2), and the y-axis represents the difference between the two measurements (Test 1 minus Test 2). The solid red line indicates the mean difference, and the dashed red lines indicate the upper and lower limits of agreement (mean ± 1.96 SD). The second assessment was completed approximately two weeks after the initial administration. Alarm Coping subscale scores reflect reverse-scored items (Items 6–9).

**Supplementary Figure S2. Scatter Plots for Convergent and Hypothesis-Specific Validity of the Japanese CAFQa.**


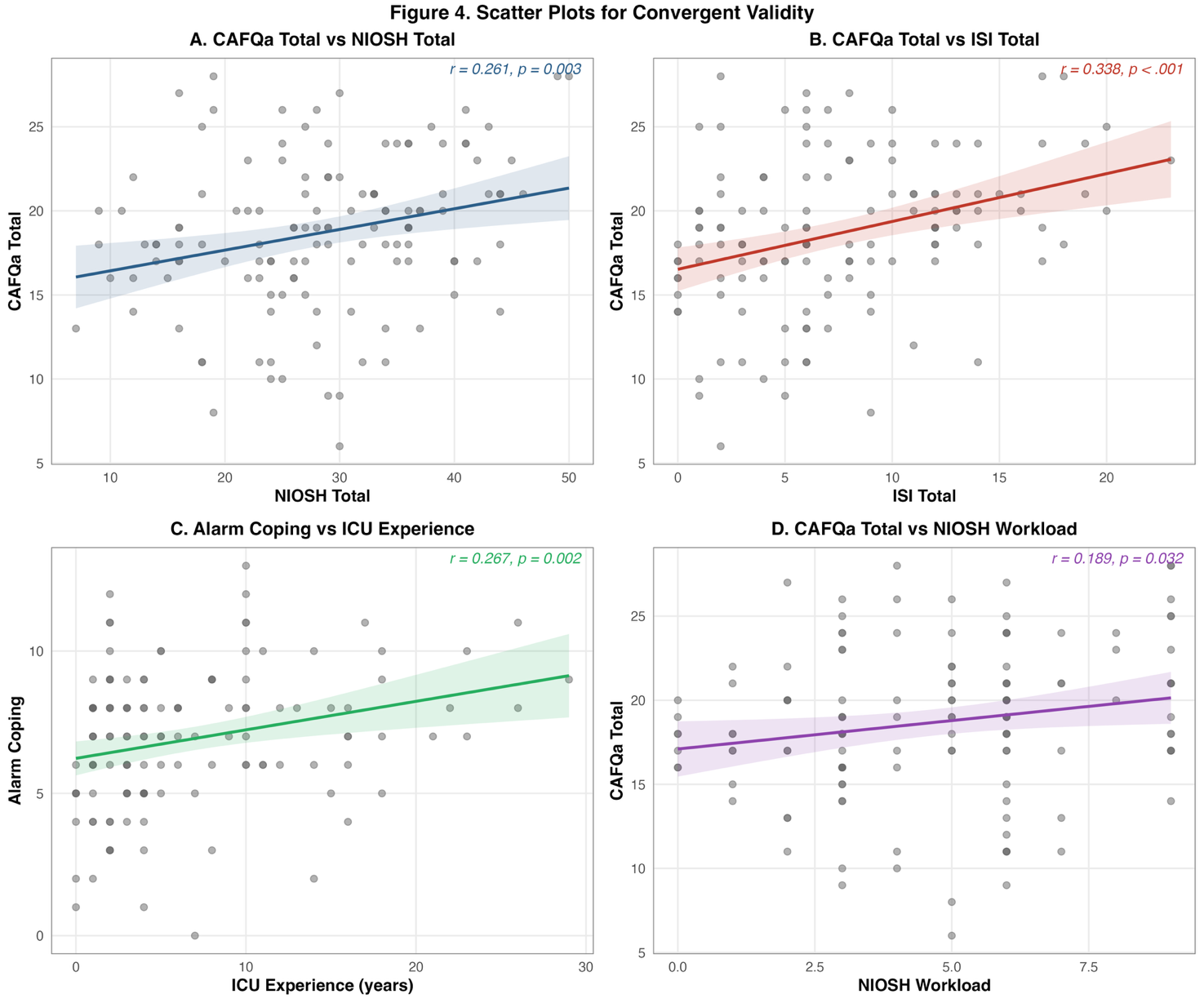
Figure legend. CAFQa = Charité Alarm Fatigue Questionnaire; NIOSH = National Institute for Occupational Safety and Health Brief Job Stress Questionnaire; ISI = Insomnia Severity Index.

Panel A shows the correlation between the CAFQa total score and the NIOSH Brief Job Stress Questionnaire total score. Panel B shows the correlation between the CAFQa total score and the Insomnia Severity Index (ISI) total score. Panel C shows the correlation between the Alarm Coping subscale score and years of ICU experience. Panel D shows the correlation between the CAFQa total score and the NIOSH quantitative workload subscale score. Each data point represents an individual participant. Solid lines indicate fitted regression lines, and shaded areas represent 95% confidence intervals. Pearson's correlation coefficients (r) and p values are displayed in each panel. Alarm Coping subscale scores reflect reverse-scored items (Items 6–9), with higher scores indicating poorer alarm coping.
